# Editorial: Insights in the minimally invasive surgery for the repair of pectus excavatum

**DOI:** 10.3389/fsurg.2023.1334043

**Published:** 2024-01-04

**Authors:** Yeung-Leung Cheng

**Affiliations:** ^1^ Department of Surgery, Taipei Tzu-Chi Hospital, Buddhist Tzu Chi Medical Foundation, New Taipei City, Taiwan; ^2^ School of Medicine, Tzu Chi University, Hualien, Taiwan

**Keywords:** pectus excavatum, minimally invasive surgery, pain control, spontaneous pneumothorax, outcomes

**Editorial on the Research Topic** Insights in the minimally invasive surgery for the repair of pectus excavatum

Pectus excavatum is considered the most common congenital chest wall deformity, for which surgical intervention is one of the standard treatments. Minimally invasive repair of pectus excavatum (MIRPE) was introduced by Nuss and his colleagues (1). The correction process involves placing a substernal stainless-steel bar across the bilateral pleural cavities using minimally invasive surgery. It adopts the concept of remodeling to correct the concave chest wall, with the bar removed after 2–4 years of correction (2). The articles in this section that are receiving ongoing attention and discussion include modification of surgery to improve correction outcomes and safety, postoperative pain control, and special thoracic diseases associated with pectus excavatum (such as spontaneous pneumothorax, etc.). These articles provide useful information and allow clinicians to gain further insight into the clinical management, surgical techniques, and postoperative care of pectus excavatum and the MIRPE.

Park et al. introduced a novel method of repairing pectus excavatum using a crane machine to elevate the depressed chest wall, eliminating the need for traditional pectus bar leverage. Out of 3,622 deformity repairs, 691 were carried out using this crane-powered technique between 2017 and 2022. The procedure includes the elevation of the sternum using a wire/screw crane, chest wall remodeling with bars, and bar fixation. Key outcomes indicate no bar displacement in any case, with minor complications including pneumothorax (7.4%), pleural effusion (1.6%), and wound issues (0.4%). Overall, the technique showed impressive results with minimal complications.

Wang et al. presented a study protocol to compare outcomes and hospitalization costs between the single-incision non-thoracoscopic Nuss procedure (SINTNP) and the thoracoscopic Nuss procedure (TNP) for pectus excavatum. This is a prospective, multicenter, randomized, controlled trial that will enroll 320 patients, aged 3–18 years without complex anomalies, who will be assigned to either the SINTNP or TNP group in a 1:1 ratio. The primary outcomes will be thoracic-related complications and hospitalization costs. The secondary outcomes focus on surgery duration and length of hospital stay. The study's focus on simple pectus deformity, specific complications, and hospitalization expenses may require a broader examination of patient outcomes for a comprehensive assessment of its benefits.

Postoperative pain control is an important issue for patients after MIRPE (3). Eldredge and McMahon investigated the use of intercostal nerve cryoablation as an analgesic strategy after MIRPE. The study, based on a literature review, suggests that intercostal nerve cryoablation results in reduced hospitalization length and opioid consumption. Although intercostal nerve cryoablation is associated with fewer short- and long-term complications, the impact on total hospital costs is variable. The research underscores the effectiveness and safety of intercostal nerve cryoablation in the management of post-operative pain in patients following surgery, with the potential for significant cost savings and shorter hospital stays.

Lee et al. addressed an intriguing connection between pectus excavatum and primary spontaneous pneumothorax. The research highlights that individuals with both conditions share common characteristics; they tend to be young, tall, and thin. Moreover, the study reveals a potential predisposing factor, the Haller Index, which measures the severity of pectus excavatum and tends to be higher in primary spontaneous pneumothorax and pectus excavatum. This underscores the importance of vigilance in monitoring both conditions together. However, the small sample size and retrospective design should be acknowledged as potential limitations, and further investigation may address these concerns. The authors recommend close observation for primary spontaneous pneumothorax even if no postoperative complications arise in patients following MIRPE.

In conclusion, these articles collectively represent strides in the field of MIRPE. They bring forth innovative techniques, associations, and pain management strategies that have the potential to improve patient care. As we continue to refine our understanding of these advancements, collaboration between researchers, surgeons, and clinicians is crucial to translating these findings into improved patient outcomes.
